# ﻿The potter wasp genus *Allorhynchium* from Vietnam, with descriptions of three new species and a new country record (Hymenoptera, Vespidae, Eumeninae)

**DOI:** 10.3897/zookeys.1166.102674

**Published:** 2023-06-06

**Authors:** Lien Thi Phuong Nguyen, Anh D. Nguyen, Ngat Thi Tran, Manh Thanh Nguyen, Michael S. Engel

**Affiliations:** 1 Insect Ecology Department, Institute of Ecology & Biological Resources (IEBR), Vietnam Academy of Science & Technology, 18 Hoang Quoc Viet Road, Nghia Do, Cau Giay, Hanoi, Vietnam Vietnam Academy of Science & Technology Hanoi Vietnam; 2 Graduate University of Science and Technology, Vietnam Academy of Science & Technology, 18 Hoang Quoc Viet Road, Nghia Do, Cau Giay, Hanoi, Vietnam University of Science and Technology Hanoi Vietnam; 3 Division of Invertebrate Zoology, American Museum of Natural History, Central Park West at 79th Street, New York, 10024-5192 New York, USA American Museum of Natural History New York United States of America; 4 Division of Entomology, Natural History Museum, and Department of Ecology and Evolutionary Biology, 1501 Crestline Drive – Suite 140, University of Kansas, Lawrence, 66045-4415 Kansas, USA University of Kansas Lawrence United States of America

**Keywords:** Biodiversity, identification key, Oriental region, solitary wasps, taxonomy

## Abstract

Species of the potter wasp genus *Allorhynchium* van der Vecht (Eumeninae: Odynerini) occurring in Vietnam are presented. Seven species have been recorded from Vietnam. Of them, three species are described as new to science: *Allorhynchiumlatum* Nguyen, Tran & MT Nguyen, **sp. nov.**, *A.moerum* Nguyen & AD Nguyen, **sp. nov.**, and *A.setosum* Nguyen & Engel, **sp. nov.**, and one species, *A.argentatum* (Fabricius, 1804), is recorded from Vietnam for the first time. An updated key to the Oriental species of the genus is presented.

## ﻿Introduction

The potter wasp genus *Allorhynchium* was established by van der Vecht (1963) for a group of Asian eumenines similar to those of the genus *Anterhynchium* de Saussure, 1863, although the two genera are apparently not closely related (Luo et al. 2022). *Allorhynchium* can be distinguished from *Anterhynchium* most notably by the propodeal dorsum raised shelf-like to the same level as the metanotum and the forewing with the prestigma approximately half the length of the pterostigma, as measured along the posterior margin, although some species of the former are now known to share the first character. Recently, several studies have been published on the genus *Allorhynchium* from India, China, and northeast Asia (Girish Kumar and Sharma 2015; Girish Kumar et al. 2016; Tan et al. 2018; Li et al. 2019; Luo et al. 2020). As a result of this work, a total of 18 species of *Allorhynchium* have been recorded from the Oriental region. Tan et al. (2018) provided a key to 16 Oriental species at that time. In Vietnam, three species have been recorded in prior studies by Giordani Soika (1986) and Nguyen et al. (2014, 2018): *A.chinense* (de Saussure, 1862), *A.lugubrinum* (Cameron, 1900), and *A.quadrimaculatum* Gusenleitner, 1997.

Based on extensive material deposited in the IEBR, Hanoi, Vietnam, a taxonomic review of *Allorhynchium* from Vietnam was undertaken and is presented here. Three new species are described and illustrated, a new country record is added, and an updated key is presented to all Oriental species in the genus.

## ﻿Materials and methods

**Figure 1.**
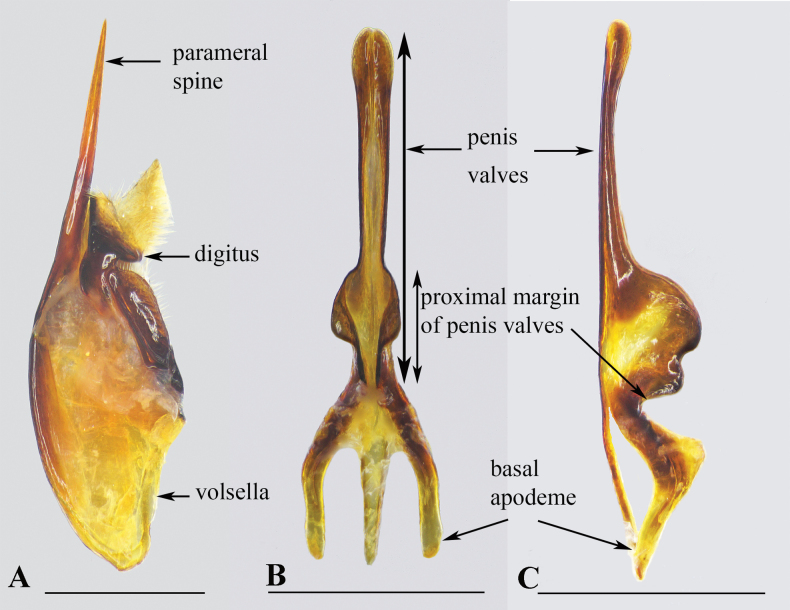
Male genitalia of *Allorhynchiumchinense* (de Saussure, 1862) **A** inner aspect of paramere with volsella and digitus **B** aedeagus, ventral view **C** aedeagus, lateral view. Scale bars: 1 mm.

Specimens of *Allorhynchium* were examined from the Insect Ecology Department, Institute of Ecology & Biological Resources (**IEBR**), Hanoi, Vietnam, and from the private collection of Seiki Yamane, Japan (**SYC**). Morphological and color characters of mature specimens were observed using pinned and dried specimens under a stereomicroscope Olympus SZ4, and measurements were made with an ocular micrometer. “Body length” indicates the combined lengths of the head, mesosoma, and the first two metasomal segments. Morphological terminology follows that of Carpenter and Cumming (1985) and Yamane (1990). Genitalic terminology follows Kojima (1999) (Fig. 1). Photographic images were made with a Nikon SMZ 800N Digital Stereo Microscope and an attached Sony α6000 digital camera. Images were stacked using Helicon Focus v.7, then grouped into a plate using Adobe Photoshop CS6. The abbreviations **F**, **S**, and **T** (I, II, III, …) refer to numbered flagellomeres, metasomal sterna, and metasomal terga, respectively. Other abbreviations are: **NP**, National Park; **NR**, National Reserve; **ISD-c**, collectors from the Insect Systematic Department (IEBR). Asterisks (*) refer to new locality records for a given species. The names of provinces in Vietnam are arranged in order from north to south and from west to east.

## ﻿Systematics

### ﻿Genus Allorhynchium

Taxon classificationAnimaliaHymenopteraEumenidae

van der Vecht, 1963

81E534D3-F3CD-549E-9B8D-F4ED12FE7996

Allorhynchium van der Vecht, 1963: 57, 58. Type species: Vespaargentata Fabricius, 1804, by original designation.

#### Diagnosis.

Anterior surface of pronotum without pits or foveae. Tegula not evenly rounded posteriorly, emarginate adjoining parategula and often shorter than latter; axillary fossa in dorsal view much narrower than long, slit-like. Metanotum flat, without tubercles. Propodeum without deep fossae, submarginal carina and valvula not protruding; propodeal dorsum raised shelf-like to same level with metanotum (but see some of the species herein that lack this feature). Mesofemur not emarginate basally. Forewing with prestigma > 1/2 length of pterostigma (as measured along posterior margin); third submarginal cell separated from apex of marginal cell by ~ 1/2 its length. Metasoma segment I sessile, ca. as wide as segment II; tergum I with tendency to form a transverse rim at junction of anterior-facing and dorsal-facing surfaces but without transverse carina.

Additionally, according to van der Vecht (1963), the declivity of the propodeum has a median carina that runs upwards into a deep, oval, fovea at a short distance from the metanotum; metasomal sternum VII of the male has one or two tubercles; and in some species metasomal sternum II is excavated or armed with two teeth or with a flattened tubercle.

### ﻿ Allorhynchium argentatum

Taxon classificationAnimaliaHymenopteraEumenidae

(Fabricius, 1804)

787AFF29-21E9-55B1-8376-007A61986D47

Fig. 2A–I

Vespa argentata Fabricius, 1804: 260.
Allorhynchium argentatum (Fabricius); van der Vecht 1963: 60.

#### Material examined.

Vietnam: 1♂, Son La, Muong La, Nam Pam, alt. 660 m, 25.vii.2009 [25 July 2009], Lien Thi Phuong Nguyen, Phong Huy Pham, J Kojima leg.; 1♀, Son La, Phu Yen, Quang Huy, 21°15'50"N, 104°38'56"E, 18.vi.2015 [18 June 2015], Dai Dac Nguyen leg.; 1♀, Son La, Yan Chau, Muong Lum, Ban Khau Khoang, 21°2'7"N, 104°26'51"E, alt. 1000 m, 20.vi.2015 [20 June 2015], Dai Dac Nguyen leg; 1♂, Son La, Moc Chau, Pha Luong, 20°43'09"N, 104°37'04"E, alt. 630 m, 22.vi.2020 [22 June 2020], Lien Thi Phuong Nguyen, Cuong Quang Nguyen, Ngat Thi Tran, Thai Van Mai leg.; 1♂, Hai Phong, Cat Ba, Cat Hai, 20°50'39"N, 106°56'42"E, 24.vii.2013 [24 July 2013], Lien Thi Phuong Nguyen, Dai Dac Nguyen leg.; 2♂♂, Hoa Binh, Mai Chau, Chieng Chau, Lac village, alt. 600 m, 10.vi.2008 [10 June 2008], Lien Thi Phuong Nguyen, Phong Huy Pham leg.; 1♀, Hoa Binh, Mai Chau, Pa Co, 21°56'16.7"N, 102°52'58.1"E 22.vii.2009 [22 July 2009], Lien Thi Phuong Nguyen, Phong Huy Pham, J Kojima leg.; 1♀, 1♂, Thanh Hoa, Quan Hoa, Pu Hu NR, 20°31'32.1"N, 104°57'38.5"E, alt. 284 m, 12.vi.2016 [12 June 2016], Lam Xuan Truong, Dai Dac Nguyen, Ngat Thi Tran, Linh Ngoc Ha leg.; 1♂, Thanh Hoa, Quan Hoa, Pu Hu NR, 20°29'13.3"N, 104°57'47.2"E, alt. 408 m, 13.vi.2016 [13 June 2016], Lam Xuan Truong, Dai Dac Nguyen, Ngat Thi Tran, Linh Ngoc Ha leg.; 1♂, Thanh Hoa, Quan Hoa, Pu Hu NR, 20°33'56"N, 104°58'39.7"E, alt. 255 m, 14.vi.2016 [14 June 2016], Dai Dac Nguyen, Ngat Thi Tran, Linh Ngoc Ha leg.; 2♂♂, Thanh Hoa, Quan Hoa, Pu Hu NR, 20°33'37.3"N, 105°00'37.9"E, alt. 120 m, 15.vi.2016 [15 June 2016], Dai Dac Nguyen, Ngat Thi Tran, Linh Ngoc Ha leg.; 5♀♀, 6♂♂, Thanh Hoa, Trung Son, 1.x.2017 [1 October 2017], Lien Thi Phuong Nguyen leg.; 1♀, Quang Tri, Dakrong, Huc Nghi, 18.vii.2004 [18 July 2004], ISD-c leg.; 1♀, Quang Tri, Huong Hoa, Huong Linh, 5.vi.2006 [5 June 2006], ISD-c leg.; 1♀, Kon Tum, Dak Ha, Dak Mar, coffee garden, alt. 603 m, 19.vii.2012 [19 July 2012], Lien Thi Phuong Nguyen leg.; 1♂, Kon Tum, Sa Thay, Chu Mom Ray NP, 31.iii–4.iv.2014 [31 March – 4 April 2014], Tru Vu Hoang leg.; 1♂, Kon Tum, Dak Glei, Dak Choong, Ngoc Linh NR, 15°11.8'N, 107°47.6'E, alt. 1064 m, 9.iv.2015 [9 April 2015], Lien Thi Phuong Nguyen, Dai Dac Nguyen, Minh Phuong Nguyen leg.; 1♀, 1♂, Kon Tum, Dak Glei, Dak Choong, Ngoc Linh NR, 15°18'N, 107°49'E, alt. 849 m, 10.iv.2015 [10 April 2015], Lien Thi Phuong Nguyen, Dai Dac Nguyen, Minh Phuong Nguyen leg.; 1♀, Gia Lai, KBang, Dak Roong, Kon Ka Kinh NP, 8.vi.2011 [8 June 2011], ISD-c leg.; 1♀, 1♂, Gia Lai, Ka Bang, Konpne, Kon Ka Kinh NP, 14°23'22.9"N, 108°20'27.5"E, alt. 847 m, 16.vii.2012 [16 July 2012], Lien Thi Phuong Nguyen leg.; 2♀♀, Gia Lai, Chu Se, rubber and pepper garden, 14.iv.2013 [14 April 2013], Lien Thi Phuong Nguyen leg.; 1♀, Dak Lak, Krong Bong, Chu Yang Sin NP, 12°27'05.3"N, 108°20'24.3"E, alt. 744 m, 5.v.2016 [5 May 2016], Lien Thi Phuong Nguyen, Dai Dac Nguyen, Ngat Thi Tran leg.; 1♂, Dak Lak, Krong Bong, Chu Yang Sin NP, 12°24'56.8"N, 108°21'02.1"E, alt. 772 m, 9.v.2018 [9 May 2018], Lien Thi Phuong Nguyen, Lam Xuan Truong, Tuan Viet Luong leg.; 3♀♀, 9♂♂ Dak Nong, Dak Glong, Dak Som, Ta Dung NR, 11°50'16.1"N, 107°59'16.7"E, alt. 745 m, 6.v.2016 [6 May 2016], Lien Thi Phuong Nguyen, Dai Dac Nguyen, Ngat Thi Tran leg.; 1♀, Dong Nai, Tan Phu, Nam Cat Tien NP, 9.viii.2005 [9 August 2005], Lien Thi Phuong Nguyen, J Kojima leg.; 1♀, Ba Ria – Vung Tau, Con Dao, Ben Dam, 29.x.2020 [29 October 2020], Hiep Duc Nguyen leg. [IEBR]. India; 1♀, Tamil Nadu, Loyola, 16–18.XII.1978 [16–18 December 1978], Madras leg. [SYC]. Indonesia: 1♂, Sako NR, Tapan, W. Sumatra, 4–5.ix.1985 [4–5 September 1985], S. Yamane leg.; 2♂, Lubuk Gadang, W. Sumatra, 22.vii.1985 [22 July 1985], Sk & S. Yamane leg. [SYC].

#### Remarks.

**Figure 2.**
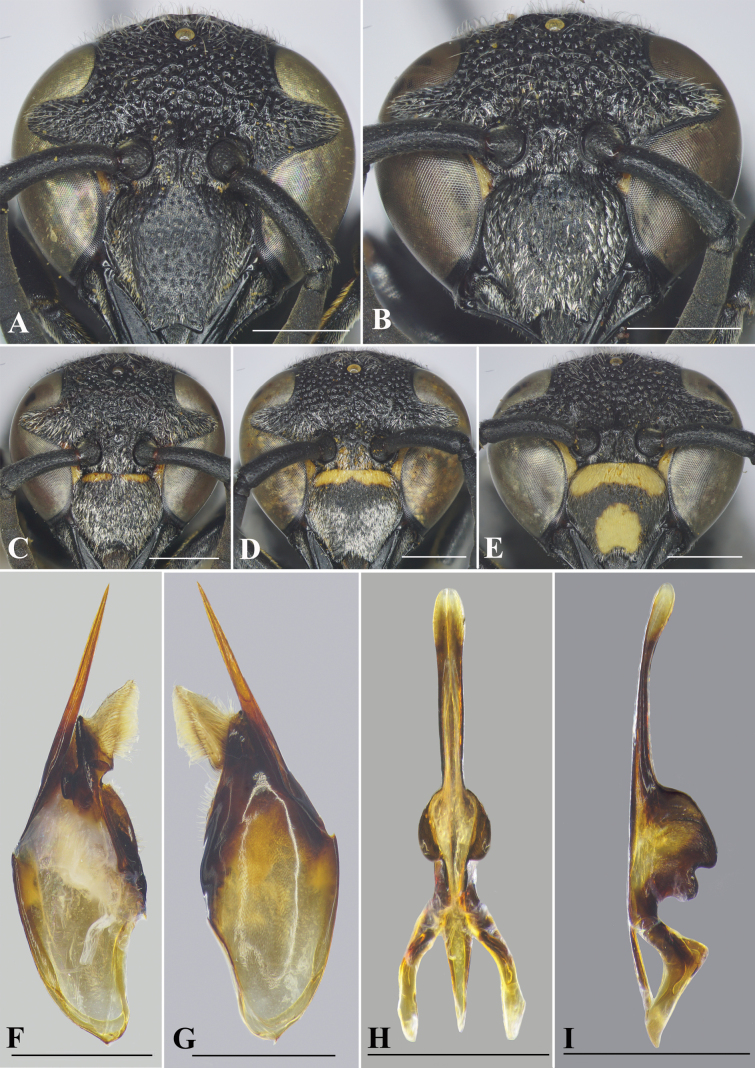
*Allorhynchiumargentatum* (Fabricius, 1804) Specimens from Vietnam **A** female, facial view **B–E** male, facial view **F** genitalia, inner aspect of paramere with volsella and digitus **G** genitalia, outer aspect of paramere with volsella and digitus. **H** aedeagus, ventral view **I** aedeagus, lateral view. Scale bars: 1 mm.

This species is recorded from Vietnam for the first time, but there is a color variation in the male clypeus: entirely black (Fig. 2B), almost black with two narrow transverse yellow strips basally (Fig. 2C), black with a transverse yellow strip basally (Fig. 2D), and black with a thick transverse yellow strip basally plus a large yellow spot medi-apically (Fig. 2E). Male genitalia of the species are described here for the first time.

#### Male genitalia.

As in Fig. 2F–I. Parameral spine lacking setae. Volsella flattened, spatulate, wide on inner aspect, and with setae at top (Fig. 2F, G). Digitus gradually widen from base to two-thirds length, then extended to apex to form two lobes, a black short one with serrated teeth laterally and a transparent lance lobe with dense long setae (Fig. 2F, G). Penis valves long, much longer than basal apodeme (~ 1.36× as long as basal apodeme); in ventral view proximal part strongly produced laterally into oval shape (Fig. 2H); in profile apical part strongly produced into two large lobes (Fig. 2I), upper lobe larger than lower lobe; proximal margin smooth (Fig. 2I).

#### Distribution.

Pakistan; India: Andaman & Nicobar Islands, Arunachal Pradesh, Assam, Chhattisgarh, Goa, Himachal Pradesh, Karnataka, Kerala, Meghalaya, Mizoram, Sikkim, Tamil Nadu, Tripura, Uttarakhand, Uttar Pradesh, West Bengal; Nepal; Bhutan; Myanmar; Thailand; Laos; Malaysia; Singapore; Indonesia: Sumatra, Java, Bali, Borneo, Sulawesi; Philippines: Mindanao; Vietnam (new record).

### ﻿ Allorhynchium chinense

Taxon classificationAnimaliaHymenopteraEumenidae

(de Saussure, 1862)

318CEAA0-39A1-5FBB-9AE3-3001CC3945F5

Rhynchium chinense de Saussure, 1862: 186.
Allorhynchium chinense (de Saussure); van der Vecht 1963: 60; Nguyen et al. 2014: 8.

#### Material examined.

Taiwan. 1♀1♂, Shihtyutou Nantou Hsien, 10.x.1996 [10 October 1996]. C.C. Luo leg.; 1♂, Shihtyutou Nantou Hsien, 8.viii.1996 [8 August 1996] [SYC].

#### Remarks.

Although Giordani Soika (1986) recorded this species from Vietnam, we have been unable to examine any specimens from the country. Despite considerable sampling of the genus from throughout Vietnam and the large number of specimens examined, we have not identified any belonging to *A.chinense*. A new species described herein as *A.setosus* (vide infra) is quite similar to *A.chinense*, as discussed under that species, and it is possible that Giordani Soika’s (1986) material belongs to that species. As we have not been able to source material from which he based his determinations, we therefore continue to list *A.chinense* here pending future work. For the moment the status of this species in Vietnam is dubious.

#### Distribution.

China: Sichuan, Yunnan, Hong Kong, Guangdong, Guangxi, Guangzhou, Macao, Jiangxi, Shanghai, Henan, Fujian; Taiwan; ? Vietnam; ? Philippines: Mindanao.

### ﻿ Allorhynchium lugubrinum

Taxon classificationAnimaliaHymenopteraEumenidae

(Cameron, 1900)

B0CFB874-8AAE-5F29-83B3-472E77582D9D

Fig. 3A, B

Rhynchium lugubrinum Cameron, 1900: 532.
Allorhynchium lugubrinum (Cameron); van der Vecht 1963: 60; Giordani Soika 1996: 37; Girish Kumar and Sharma 2015: 21; Girish Kumar et al. 2016: 30.

#### Material examined.

Vietnam: 1♀, Ha Giang, Yen Minh, Yen Minh town, 23°07'06"N, 105°08'20"E, 30.ix.2014 [30 September 2014], Lam Xuan Truong, Lien Thi Phuong, Minh Phuong Nguyen, Dai Dac Nguyen leg.; 1♀1♂, Tuyen Quang, Na Hang, 22°20'52.6"N, 105°25'49"E, alt. 120 m, 10.vi.2015 [10 June 2015], Lien Thi Phuong Nguyen, Dai Dac Nguyen, Lam Xuan Truong leg.; 1♀, Son La, Thuan Chau, Copia NP, alt. 600 m, 4.vi.2009 [4 June 2009], Phong Huy Pham leg.; 1♀, Son La, Moc Chau, Pha Luong, 20°43'09"N, 104°37'04"E, alt. 630 m, 22.vi.2020 [22 June 2020], Lien Thi Phuong Nguyen, Cuong Quang Nguyen, Ngat Thi Tran, Thai Van Mai leg; 1♂, Hoa Binh, Lac Thuy, Thanh Nong, 5.viii.2017 [5 August 2017], Phong Huy Pham leg. [IEBR].

**Figure 3.**
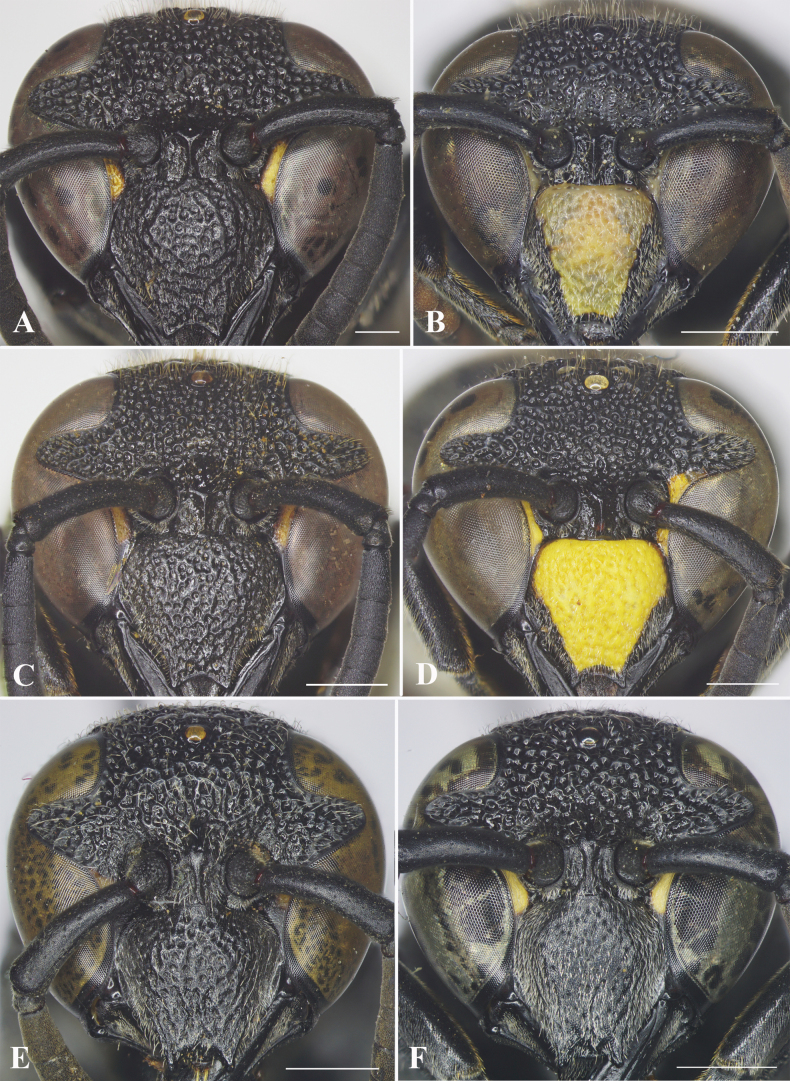
Facial views of species of *Allorhynchium*. *A.lugubrinum* (Cameron) **A** female **B** male; *A.quadrimaculatum* Gusenleitner **C** female **D** male; *A.snelleni* (Saussure) **E** female **F** male. Scale bars: 1 mm.

#### Distribution.

India: Meghalaya, Sikkim; China: Yunnan; Vietnam: Ha Giang (*), Tuyen Quang, Son La (*), Hoa Binh (*).

### ﻿ Allorhynchium quadrimaculatum

Taxon classificationAnimaliaHymenopteraEumenidae

Gusenleitner, 1997

8F2BE56C-C9B9-5A7E-BF45-DBDFCB2F6297

Fig. 3C, D

Allorhynchium quadrimaculatum Gusenleitner, 1997: 759; Gusenleitner 2011: 1362; Girish Kumar et al. 2016: 34; Tan et al. 2018: 57.

#### Material examined.

Vietnam: 1♂, Cao Bang, Nguyen Binh, Thanh Cong, 22°32'29.7"N, 105°52'51.7"E, 8.viii.2012 [8 August 2012], J Kojima, H Nugroho & IED-c leg.; 1♀, Cao Bang, Thanh Cong Nguyen Binh, Thanh Cong, Phia Oac, near commune people’s committee, 11.viii.2012 [11 August 2012], ISD-c leg.; 1♀, Cao Bang, Phia Oac, 22°35'34.4"N, 105°51'25.1"E, alt. 1035 m, 7–10.v.2013 [7–10 May 2013], Tru Van Hoang leg.; 1♂, Yen Bai, Van Chan, Thuong Bang La, 21°27'03.2"N, 104°44'23.7"E, alt. 176 m, 11.ix.2017 [11 September] 2017, Lien Thi Phuong Nguyen, Cuong Quang Nguyen, Tam Thi Thanh Vu leg.; 1♀, Tuyen Quang, Ham Yen, Phu Luu, Cham Chu NR, alt. 200 m, 13.iv.2011 [13 April 2011], Lien Thi Phuong Nguyen leg.; 1♂, Bac Kan, Na Ri, An Tinh, Kim Hy NR, 22°12'31"N, 106°5'2"E, 3.vi.2014 [3 June 2014], Lien Thi Phuong Nguyen, Dai Dac Nguyen, Duong Dinh Tran leg.; 1♀, Bac Kan, Cho Moi, Quang Chu, 21°49'50.6"N, 105°47'21.9"E, alt. 120 m, 2.iv.2018 [2 April 2018], Lien Thi Phuong Nguyen, Cuong Quang Nguyen, Ngat Thi Tran, Tuan Viet Luong leg.; 1♀; Phu Tho, Xuan Son NP, alt. 300–400 m, 24.ix.2005 [24 September 2005], Lien Thi Phuong Nguyen leg.; 1♂, Vinh Phuc, Me Linh, buffalo farm, alt. 100 m, 27.v.2000 [27 May 2000], Lien Thi Phuong Nguyen leg.; 1♀, Vinh Phuc, Tam Dao, alt. 800 m, 12.v.2003 [12 May 2003], Lien Thi Phuong Nguyen leg.; 1♀, Vinh Phuc, Phuc Yen, Me Linh Biodiversity station, 2.vi.2018 [2 June 2018], Cuong Quang Nguyen leg.; 1♀, Bac Giang, Son Dong, Tuan Dao, Khe Dan, 4.vii.2010 [4 July 2010], Duong Dinh Tran leg.; 1♀, Bac Giang, Son Dong, near Thanh Son town, alt. 120 m, 5.vii.2012 [5 July 2010], Phong Huy Pham leg.; 1♀, Bac Giang, Tay Yen Tu, Dong Thong, 12.vi.2016 [12 June 2016], Tuan Van Nguyen leg.; 1♂, Ninh Binh, Cuc Phuong NP, 8.v.2002 [8 May 2002], Tru Vu Hoang leg.; 2♂♂, Ninh Binh, Nho Quan, Cuc Phuong NP, 20°21'06"N, 105°35'23"E, alt. 482 m, 7.viii.2019 [7 August 2019], Lien Thi Phuong Nguyen, Cuong Quang Nguyen leg.; 1♂, Hoa Binh, Yen Thuy, Da Phuc, 3.v.2002 [3 May 2002], Tru Vu Hoang leg.; 1♂, Hoa Binh, Tan Lac, Ngo Luong, Ngoc Son NR, 20°24'35.4"N, 105°21'54"E, alt. 289 m, 28.viii.2020 [28 August 2020], Ngat Thi Tran leg.; 2♂♂, Thanh Hoa, Quan Hoa, Pu Hu NR, 20°29'13.3"N, 104°57'47.2"E, alt. 408 m, 13.vi.2016 [13 June 2016], Lam Xuan Truong, Dai Dac Nguyen, Ngat Thi Tran, Linh Ngoc Ha leg.; 1♂, Thua Thien Hue, A Luoi, A Roang, 3.v.2005 [3 May 2005], ISD-c leg.; 1♀, 4♂♂, Gia Lai, KBang, Konpne, Kon Ka Kinh NP, 14°23'22.9"N, 108°20'27.5"E, alt. 847 m, 15.vii.2012 [15 July 2012], Lien Thi Phuong Nguyen leg. [IEBR].

#### Remarks.

In Vietnam, this species has two color variations of the metasomal segments: one variation is wholly black except for yellow marks at the lateral sides of tergum II, and the other is black with narrow orange bands at the apical margins of terga I and II. Among our material, the latter occurs only in one male, and in this male metasomal sternum II is slightly produced subbasally rather than more or less produced to become tubercules as in other males.

#### Distribution.

China: Guangxi, Sichuan, Guizhou, Yunnan; Laos; Vietnam: Cao Bang (*), Tuyen Quang, Yen Bai (*), Bac Kan (*), Vinh Phuc, Bac Giang (*), Phu Tho (*), Hoa Binh (*), Ninh Binh (*), Thanh Hoa (*), Thua Thien Hue (*), Gia Lai (*).

### ﻿ Allorhynchium snelleni

Taxon classificationAnimaliaHymenopteraEumenidae

(de Saussure, 1862)

935121C1-35A6-5EED-AEB5-877705C140CE

Fig. 3E, F

Rhynchium snelleni de Saussure, 1862: 185.
Allorhynchium snelleni (Saussure); van der Vecht 1963: 59; Nguyen et al. 2014: 8; Girish Kumar et al. 2016: 34; Tan et al. 2018: 52.

#### Material examined.

Vietnam: 1♂, Soc Trang, My Tu, My Phuoc, 09°34'11.8"N, 105°44'52.9"E, 4.iv.2018 [4 April 2018], Hoa Thi Dang leg. [IEBR]; 1♀, Kien Giang, U Minh Thuong, 1.xii.2003 [1 December 2003], Lien Thi Phuong Nguyen leg.

#### Remarks.

This species has been recorded in Kien Giang, Vietnam by Nguyen et al. (2014). In this study one new locality is recorded.

#### Distribution.

Vietnam: Soc Trang (*), Kien Giang; Indonesia: Sumatra (including Bangka, Biliton, Pulau Sangijang, Sunda Straits), Java (including Karimunjawa, Bawean, Kangean), Kalimantan.

### ﻿ Allorhynchium latum

Taxon classificationAnimaliaHymenopteraEumenidae

Nguyen, Tran & MT Nguyen sp. nov.

488FDCBA-8867-53E8-AA5A-4D0B9D5CF15A

https://zoobank.org/9821D569-856A-4BE0-94CC-715123DA7D5F

Figs 4 , 5

#### Material examined.

***Holotype*.** Vietnam: ♀, Thua Thien Hue, Bach Ma, 16°13'N, 107°51'E, 13.viii.2005 [13 August 2005], Lien Thi Phuong Nguyen, J Kojima leg. [IEBR].

***Paratypes*.** Vietnam: 1♀, Nghe An, Con Cuong, Thac Kem, Pu Mat NP, 22°47'36"N, 104°36'44"E, alt. 280 m, 30.viii.2020 [30 August 2020], Ngat Thi Tran leg.; 1♂, Thua Thien Hue, Bach Ma, alt. 900 m, 15.viii.2005 [15 August 2005], Lien Thi Phuong Nguyen, J Kojima leg.; 1♀, Quang Nam, Phuoc Son, Phuoc My, alt. 450 m, 2.v.2005 [2 May 2005], ISD-c leg.; 1♀, Quang Nam, Phuoc Son, Phuoc My, Lo Xo pass, alt. 670 m, 28.vii.2004 [28 July 2004], ISD-c leg.; 1♀, Quang Nam, Cha Val, 29.vi.2008 [29 June 2008], ISD-c leg.; 1♀, Quang Nam, Song Thanh, Cha Va, alt. 400–600 m, 29.iv.2005 [29 April 2005], ISD-c leg.; 1♀, Quang Nam, Song Thanh, Cha Va, alt. 500–600 m, 29.iv.2005 [29 April 2005], leg.; 1♂, Quang Nam, Song Thanh, Phuoc Xuan, alt. 300–400 m, 1.v.2005 [1 May 2005], ISD-c leg. [IEBR].

#### Diagnosis.

This species can be distinguished from other species in the genus by the following combination of characters: head transverse, much wider than high, vertex of female with a small cephalic fovea bearing a tuft of setae medio-dorsally; occipital carina weakly widened laterally; inner compound eye margins strongly convergent ventrally, in facial view 1.3× further apart from each other at vertex than at clypeus; clypeus in frontal view almost as wide as high, apical margin shallowly emarginate medially, forming blunt tooth on each side, distance between teeth > 1/2 width of clypeus between inner compound eye margins; mesoscutum almost as long as wide between tegulae; propodeal dorsum raised shelf-like to same level with metanotum; SVII of male with a raised flat area basally, apical margin of flat area V-shaped. Digitus with two lobes at apex, a dark brown short one and an orange-yellow lance lobe with short hairs; penis valves slightly shorter than the basal apodeme, in profile apical part strongly produced into two lobes, the above lobe round, and larger, the below lobe much smaller and sharper.

#### Description.

**Figure 4.**
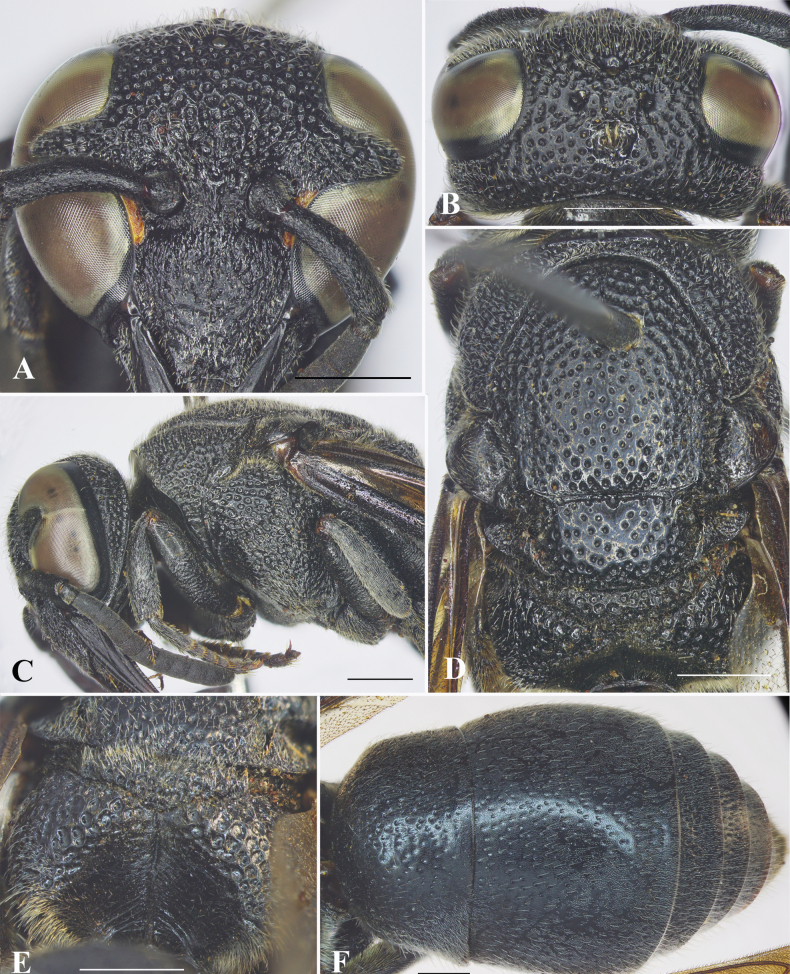
*Allorhynchiumlatum* Nguyen, Tran & MT Nguyen, sp. nov., female holotype **A** facial view **B** head, dorsal view **C** head and mesosoma, lateral view **D** mesosoma, dorsal view **E** propodeum, posterior view **F** metasoma, dorsal view. Scale bars: 1 mm.

**Female** (Fig. 4): Body length 10.6–12.1 mm (holotype = 10.6 mm); forewing length 10.1–11.6 mm (holotype = 10.1 mm).

***Structure*.** Head in facial view transverse, 1.1× as wide as high (Fig. 4A). Vertex with a small cephalic fovea bearing a tuft of setae medio-dorsally (Fig. 4B). Distance from posterior ocelli to apical margin of vertex 1.8× distance from posterior ocellus to inner compound eye margin (Fig. 4B). Gena narrower than compound eye, ~ 0.8× as wide as compound eye; occipital carina complete, present along entire length of gena, weakly widened laterally (Fig. 4C). Inner compound eye margins strongly convergent ventrally, in anterior view 1.3× further apart from each other at vertex than at clypeus (Fig. 4A). Clypeus in lateral view gradually convex in basal half, then straight to apical margin; in frontal view almost as wide as high (Fig. 4A), with basal margin slightly convex medially and distinctly separated from antennal toruli; apical margin shallowly emarginate medially, forming blunt tooth on each side, distance between teeth > ½ width of clypeus between inner compound eye margins (~ 0.6× width of clypeus between inner compound eye margins). Mandible quadridentate, teeth prominent. Antennal scape ~ 3.8× as long as its maximum width, slightly curved; FI ~ 1.3× longer than wide, FII–III almost as long as wide, FIV–IX wider than long, terminal flagellomere bullet-shaped, ca. as long as its basal width. Mesosoma longer than wide in dorsal view (Fig. 4D). Pronotal carina strongly raised, reaching ventral corner of pronotum. Mesoscutum weakly convex, almost as long as wide between tegulae, without depressed and oblique furrows apically (Fig. 4D). Disc of mesoscutellum convex, in lateral view at same level of mesoscutum (Fig. 4C), narrowly depressed basally (Fig. 4D). Metanotum weakly convex. Propodeal dorsum raised shelf-like to same level with metanotum; declivity of propodeum with median carina running upward into a narrow and long fovea at a short distance from metanotum, concavity deep and wide. Forewing with third submarginal cell separated from apex of marginal cell by < ½ its length. Metasomal segment I as wide as segment II (Fig. 4F), rounded at base; TI in dorsal view ~ 1.1× as wide as long; TII wider than long, ~ 1.1× as wide as long in dorsal view; SII depressed at base, in lateral view almost straight from base to midlength, then straight to apical margin.

***Sculpturing*.** Clypeus rugose, interspaces between punctures with minute punctures, each puncture bearing a medium-long bristle. Frons densely covered with coarse flat-bottom punctures, interspaces between punctures narrow and raised to form reticulation. Vertex with coarse punctures, punctures equal in size, interspaces with sparse minute punctures; gena with punctures similar to those on vertex from midlength to vertex, punctures smaller and less coarse in basal half (Fig. 4C); occipital carina weakly widened laterally (Fig. 4C). Pronotum with punctures similar to those on vertex. Mesoscutum covered with flat-bottomed punctures, punctures equal in size, smaller than those on pronotum, interspaces between punctures with sparse minute punctures, smooth, larger than puncture diameter centrally, punctures at margins stronger and larger than those centrally; mesoscutellum with punctures similar to those on mesoscutum, punctures on metanotum denser than those on mesoscutellum, interspaces between punctures narrow and raised to form reticulation. Mesepisternum with flat-bottomed punctures, punctures coarser to those on pronotum posterodorsally, smooth anteroventrally; border between posterodorsal and anteroventral parts distinct, without epicnemial carina. Dorsal part of metapleuron largely smooth and with several short and weak striae, ventral part with sparse and shallow punctures. Propodeum with very coarse and dense punctures dorsally, punctures much shallower and weaker to form weak transverse striae laterally, interspaces between punctures strongly raised to form reticulation, dorso-lateral margin of propodeum somewhat rounded; posterior surface rugose basally and with some short oblique striations near median carina apically. Tegula with minute punctures. Metasomal TI covered with sparse and strong punctures dorsally, fine punctures dorso-anteriorly, distance between punctures greater than puncture diameter, interspaces with minute punctures; punctures on TII smaller and shallower than those on TI, punctures on TIII–V and SII denser than those on TII; TVI and SVI with minute punctures.

***Color*.** Body almost black except two short yellow bands along inner orbits near clypeus and brown valvulae. Wing infuscate, veins dark brown (Fig. 8D).

***Pubescence*.** Body with medium-length silver setae.

**Figure 5.**
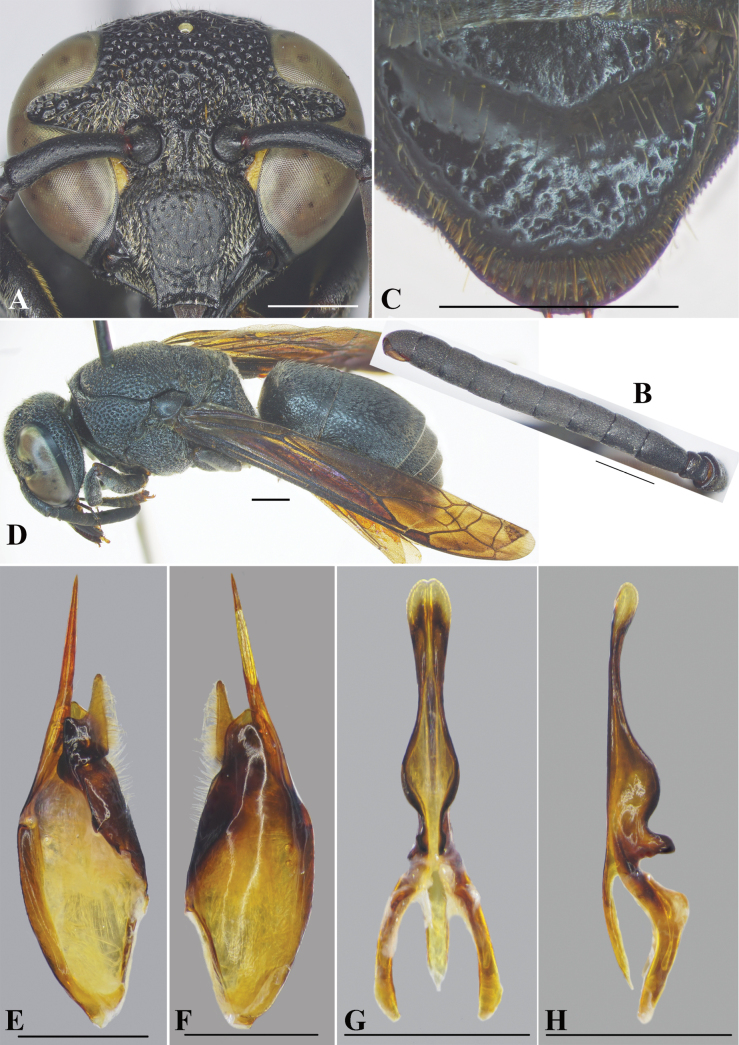
*Allorhynchiumlatum* Nguyen, Tran & MT Nguyen, sp. nov., male paratype, female holotype **A** male, facial view **B** male, antenna **C** male, metasomal sternum VII **D** female, dorsolateral oblique habitus **E** male genitalia, inner aspect of paramere with volsella and digitus **F** male genitalia, outer aspect of paramere with volsella and digitus **G** aedeagus, ventral view **H** aedeagus, lateral view. Scale bars: 1 mm.

**Male** (Fig. 5). Body length 11.9–12.3 mm; forewing length 11.4–11.7 mm.

***Structure*.** As in female but differing as follows: head transverse, much wider than high, 1.2× as wide as high in facial view (Fig. 5A); vertex without cephalic foveae; distance from posterior ocelli to apical margin of vertex ~ 2.2× distance from posterior ocelli to inner compound eye margin; inner compound eye margins strongly convergent ventrally, in facial view 1.2× as further apart from each other at vertex as at clypeus; clypeus in frontal view as wide as long, apical margin slightly emarginate medially, forming blunt tooth on each side (Fig. 5A), width of emargination much greater than one-third of clypeal width between inner compound eye margins (0.4× width of clypeus between inner compound eye margins); mandible with short teeth. Antennal scape ~ 4.45× as long as wide, FI ~ 1.5× as long as wide, FII–IV longer than wide, FV–VII as wide as long, FVIII wider than long, FIX slightly longer than wide, FX much smaller than FIX, terminal flagellomere elongate, slightly curved, ~ 3× as long as its basal width (Fig. 5B). Metasomal SVII with a raised flat area basally, apical margin of flat area V-shaped (Fig. 5C).

***Sculpturing*.** Body surface sculptured as in female; clypeus with sparse and small punctures, distance between punctures greater than puncture diameter and with dense minute punctures.

***Pubescence*.** As in female except clypeus covered with dense, medium-long, silver setae laterally.

***Color*.** Similar to female.

***Genitalia*.** As in Fig. 5E–H. Parameral spine lacking hairs. Volsella flattened, spatulate, wide on inner aspect, and without hairs at top (Fig. 5E, F). Digitus gradually widened from base to two-thirds length, then extended apically to form two lobes, a dark brown short one and an orange-yellow lance lobe with short hairs (Fig. 5E, F). Penis valves long, ~ 1.7× as long as basal apodeme, in ventral view proximal part produced laterally into an oval shape (Fig. 5G); in profile apical part strongly produced into two lobes (Fig. 5H), upper lobe round and larger, lower lobe much smaller and sharper; proximal margin smooth (Fig. 5H).

#### Distribution.

Central Vietnam.

#### Etymology.

The specific epithet is from the Latin participle *lātus* (meaning broad or wide), and refers to the wide apical emargination of the clypeus.

#### Remarks.

The new species is similar to *A.argentatum* in that all have the occipital carina slightly widened laterally, the propodeal dorsum raised shelf-like to the same level of the metanotum, the posterior surface of the propodeum rugose basally and with some short oblique striations near the median carina apically, TI finely punctate anteriorly, and TII with small and sparse punctures. However, it differs from *A.argentatum* in the following characters: head transverse, apical margin of clypeus with wide emargination, > ½ and > 1/3 width of clypeus between inner compound eye margins in female and male, respectively (~ 1/3 width of clypeus between inner compound eye margins in both female and male in *A.argentatum*); mandible of male with short teeth (mandible of male with prominent teeth in *A.argentatum*); SVII of male with apical margin of raised flat area V-shaped (SVII with apical margin of raised flat area wide in *A.argentatum*); penis valves short, slightly shorter than the basal apodeme (penis valves long, much longer than the basal apodeme in and *A.argentatum*); proximal part of aedeagus in profile with two lobes, the below lobe small and sharp (proximal part of aedeagus in profile with two lobes, the below lobe much larger and blunter in *A.argentatum*).

### ﻿ Allorhynchium moerum

Taxon classificationAnimaliaHymenopteraEumenidae

Nguyen & AD Nguyen sp. nov.

AFA3BDE8-64A9-5353-887C-87CA40F0D1C9

https://zoobank.org/6C7D8C16-BE52-479C-896E-B40B79E79759

Figs 6 , 7

#### Material examined.

***Holotype*.** Vietnam: ♀, Thanh Hoa, Thuong Xuan, Van Xuan, Hoan Can, Xuan Lien NR, 19°52'27.5"N, 105°14'20.8"E, alt. 106 m, 24.viii.2012 [24 August 2012], Lien Thi Phuong Nguyen leg. [IEBR].

***Paratypes*.** Vietnam: 1♂, Ha Giang, Yen Minh, Du Gia, Khan Ria, 30.iv.2000 [30 April 2000], Lien Thi Phuong Nguyen leg.; 1♀, Ha Giang, Bac Me, Lung Cang, 22°43'17.1"N, 105°11'43.8"E alt. 219 m, 20.vii.2019 [20 July 2019], Cuong Quang Nguyen, Hoa Thi Dang, Thai Van Mai leg.; 2♂♂, same data as holotype; 2♂♂, Cao Bang, Nguyen Binh, Thanh Cong, 22°32'29.7"N, 105°52'51.7"E, 8.viii.2012 [8 August 2012], J Kojima, H Nugroho & IED-c leg.; 2♀♀, Lang Son, Huu Lung, Huu Lien, Lan Chau, Huu Lien NR, 12.vi.2018 [12 June 2018], alt. 370 m, Lien Thi Phuong Nguyen, Lam Xuan Truong, Ngat Thi Tran, Tuan Viet Luong, Ha Thi Thu Nguyen leg.; 1♀, Vinh Phuc, Me Linh, buffalo farm, alt. 100 m, 24.v.2000 [24 May 2000], Lien Thi Phuong Nguyen leg.; 1♀, Vinh Phuc, Me Linh, buffalo farm, alt. 100 m, 27.v.2000 [27 May 2000], Lien Thi Phuong Nguyen leg.; 1♀, Thai Nguyen, Phu Luong, Yen Do, 19.x.2015 [19 Octorber 2015], Lien Thi Phuong Nguyen, Dai Dac Nguyen, Minh Phuong Nguyen leg.; 1♀, Bac Giang, Son Dong, Tay Yen Tu, alt. 200–300 m, 3.vii.2010 [3 July 2010], Phong Huy Pham leg.; 1♀, Quang Ninh, Hoanh Bo, Dong Quang, 2.viii.2013 [2 August 2013], Tuan Van Nguyen leg.; 1♀, Phu Tho, Xuan Dai, Tan Son, 19.v.2011 [19 May 2011], Phong Huy Pham leg.;1♀, Hanoi, Hoa Duc, An Khanh, 21.viii.2015 [21 August 2015], Minh Phuong Nguyen leg.; 1♀, Hanoi, Thach That, Dong Truc, 26.viii.2015 [26 August 2015], Dai Dac Nguyen leg.; 1♂, Thanh Hoa, Thuong Xuan, Van Xuan, Hoan Can, Xuan Lien NR, 19°51'41.2"N, 105°14'06.6"E, alt. 175 m, 23.viii.2012 [23 August 2012], Lien Thi Phuong Nguyen leg.; 1♂, Ha Tinh, Huong Khe, Son Kim, 6.v.2004 [6 May 2004], Lam Xuan Truong leg.; 1♀, Thua Thien Hue, Bac Ma NP, alt. 400 m, 15.viii.2005 [15 August 2005], Lien Thi Phuong Nguyen, J Kojima leg.; 1♂, Thua Thien Hue, Bac Ma NP, alt. 50 m, 16°15'N, 107°52'E, 16.viii.2005 [16 August 2005], Lien Thi Phuong Nguyen, J Kojima leg. [IEBR].

#### Other material examined.

1♂, Cao Bang, Ha Lang, 24.vii.2015 [24 July 2015], Tuan Van Nguyen leg.; 1♀, Cao Bang, Nguyen Binh, Tran Hung Dao forest, 22°36'17"N, 106°01'47.6"E, alt. 470 m, 18.x.2015 [18 October 2015], Lien Thi Phuong Nguyen, Dai Dac Nguyen, Minh Phuong Nguyen leg.; 1♀, Dien Bien, 21°56'16.7"N, 102°52'58.1"E, 22.vii.2009 [22 July 2009], Lien Thi Phuong Nguyen, Phong Huy Pham, J Kojima leg.; 1♂, Lao Cai, Lao Cai City, 20.vi.2008 [20 June 2008], Lien Thi Phuong Nguyen, Phong Huy Pham leg.; 1♀, Tuyen Quang, Na Hang, Na Hang NR, Road to Ban Bung, 22°16'59.5"N, 105°26'01"E, alt. 369 m, 11.vi.2015 [11 June 2015], Lien Thi Phuong Nguyen, Dai Dac Nguyen, Lam Xuan Truong leg.; 1♀, Lang Son, Huu Lung, Cai Kinh, 22°39'42.9"N, 106°15'36"E, alt. 28 m, 24.xi.2015 [24 November 2015], Lien Thi Phuong Nguyen, Dai Dac Nguyen, Ngat Thi Tran leg.; 2♀♀, 1♂, Lang Son, Huu Lung, Cai Kinh, 20°31'37.6"N, 105°00'24.2"E, alt. 86 m, 16.vii.2016 [16 July 2016], Lien Thi Phuong Nguyen, Dai Dac Nguyen, Ngat Thi Tran leg.; 2♀♀, Vinh Phuc, Me Linh, Me Linh Station, 13–14.v.2013 [13–14 May 2013], Lien Thi Phuong Nguyen leg.; 1♂, Hoa Binh, Yen Thuy, Lac Thinh, 2.v.2002 [2 May 2002], Tru Hoang Vu leg.; 2♂♂, Hoa Binh, Yen Thuy, Da Phuc, 4.v.2002 [4 May 2002], Tru Vu Hoang leg.; 1♂, Hoa Binh, Yen Thuy, Lac Thinh, 6.v.2002 [6 May 2002], Tru Hoang Vu leg.; 1♀, Hanoi, Ba Vi NP, alt. 700 m, 14.viii.2000 [14 August 2000], ISD-c leg.; 1♀, Hanoi, Ba Vi, Yen Bai, Mo stream, alt. above 100 m, 1.vi.2001 [1 May 2001], Lien Thi Phuong Nguyen leg.; 1♀, Hanoi, Long Binh, Tu Dinh, 16.vii.2011 [16 July 2011], Hoa Thi Dang leg.; 1♂, Hanoi, Ba Vi, Da Chong, 25.viii.2013 [25 August 2013], Dai Dac Nguyen leg. [IEBR]

#### Diagnosis.

This species can be distinguished from other species in the genus by the following character combination: head of female in facial view transverse, 1.2× as wide as high; vertex of female produced behind ocelli, with small cephalic foveae situated next to each other and each bearing a tuft of setae; clypeus in frontal view slightly wider than high, apical margin shallowly emarginate medially, forming blunt tooth on each side, distance between teeth slightly > 1/3 width of clypeus between inner compound eye margins; mesoscutum slightly shorter than wide; propodeal dorsum raised shelf-like to same level with metanotum, concavity deep and wide, margined by a crest, posterior surface clearly separated from dorsal and lateral surfaces by a curved rim, rim strongly produced behind metanotum to form serrate teeth; SVII of male with raised flat area basally, apical margin of raised flat area wide; volsella with long setae at the top.

#### Description.

**Figure 6.**
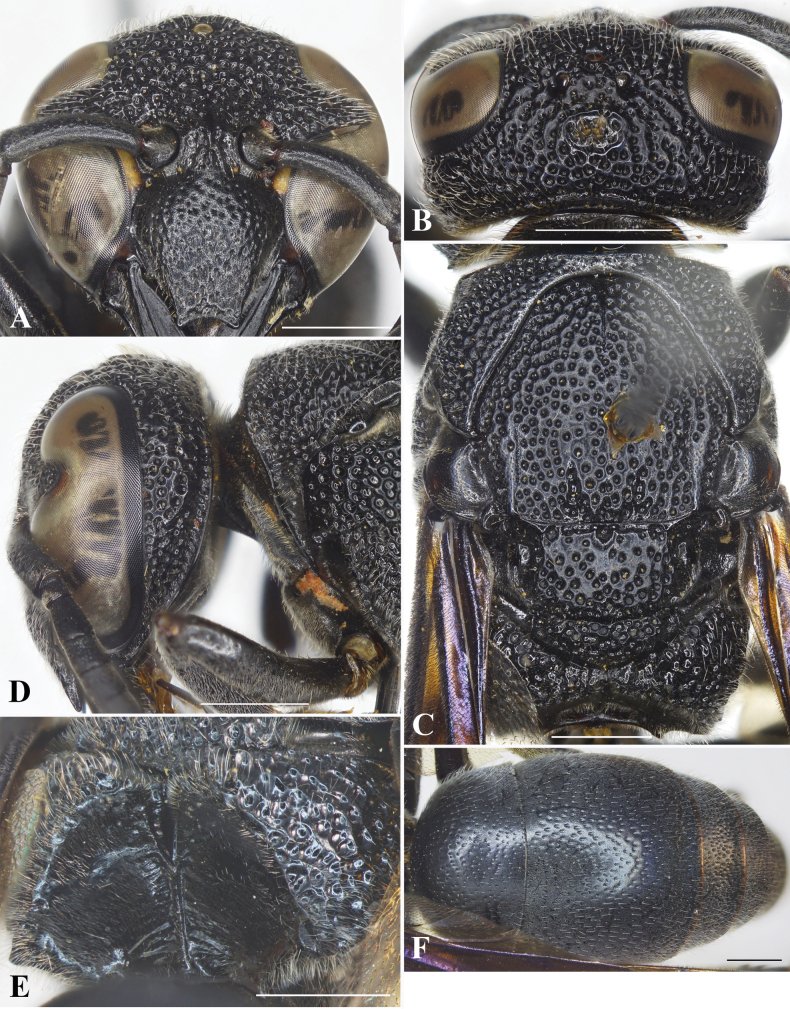
*Allorhynchiummoerum* Nguyen & AD Nguyen, sp. nov., female holotype **A** facial view **B** head, dorsal view **C** head and mesosoma, lateral view **D** mesosoma, dorsal view **E** propodeum, posterior view **F** metasoma, dorsal view. Scale bars: 1 mm.

**Female** (Fig. 6): Body length 12.3–13.1 mm (holotype = 12.3 mm); forewing length 11.0–12.1 mm (holotype = 11.0 mm).

***Structure*.** Head in anterior view subcircular, 1.1 × as wide as high (Fig. 6A). Vertex with small cephalic foveae situated next to each other, each bearing a tuft of setae (Fig. 6B). Distance from posterior ocelli to apical margin of vertex 2.3× distance from posterior ocellus to inner compound eye margin (Fig. 6B). Gena narrower than compound eye, ~ 0.86× as wide as compound eye; occipital carina complete, present along entire length of gena, widened laterally. Inner compound eye margins strongly convergent ventrally, in facial view 1.2× further apart from each other at vertex than at clypeus. Clypeus in lateral view prominently convex in basal half, then straight to apical margin; in frontal view slightly wider than high, ~ 1.04× as wide as high (Fig. 6A), with basal margin slightly convex medially and distinctly separated from antennal toruli; apical margin shallowly emarginate medially, forming blunt tooth on each side, distance between teeth slightly > 1/3 width of clypeus between inner compound eye margins (~ 0.4× width of clypeus between inner compound eye margins). Mandible quadridentate. Antennal scape ~ 4.3× as long as its maximum width, slightly curved; FI ~ 1.4× longer than wide, FII slightly longer than wide, FIII–IX wider than long, terminal flagellomere bullet-shaped, ca. as long as its basal width. Mesosoma longer than wide in dorsal view (Fig. 6C). Pronotal carina slightly raised, reaching ventral corner of pronotum. Mesoscutum weakly convex, slightly shorter than wide, 0.95× as long as wide between tegulae, with two depressed and oblique furrows running from apical margin to one-fifth length of mesoscutum (Fig. 6C). Disc of mesoscutellum almost flat, in lateral view at same level of mesoscutum, depressed basally (Fig. 6C). Metanotum weakly convex. Declivity of propodeum with median carina running upward into a deep, oval, fovea at a short distance from metanotum, propodeal dorsum raised shelf-like to same level with metanotum, concavity deep and wide, margined by a crest, posterior surface clearly separated from dorsal and lateral surfaces by a curved rim, rim strongly produced behind metanotum to form serrate teeth (Fig. 6E). Forewing with third submarginal cell separated from apex of marginal cell by < ½ its length. Metasomal segment I (Fig. 6F) as wide as segment II, rounded at base; TI in dorsal view ~ 1.7× as wide as long; TII longer than wide, ~ 1.1× as long as wide in dorsal view; SII depressed at base, in lateral view almost straight from base to midlength, then straight to apical margin.

***Sculpturing*.** Clypeus with strong punctures, punctures on flattened part larger and shallower, interspaces between punctures with minute punctures, each puncture bearing a very short bristle. Frons densely covered with coarse flat-bottom punctures, interspaces between punctures with sparse minute punctures and raised to form reticulation. Vertex with punctures slightly smaller than those on frons, interspaces larger than on frons; gena from midlength to vertex with punctures similar to those on vertex, and with smaller and weaker punctures on apical half (Fig. 6D); occipital carina weakly widened laterally (Fig. 6D). Pronotum with punctures similar to those on vertex. Mesoscutum densely and coarsely covered with flat-bottomed punctures, punctures equal in size, smaller than those on pronotum, interspaces between punctures raised to form reticulation; mesoscutellum with coarse, sparser, and smaller punctures than those on mesoscutum, several small impunctate areas between coarse punctures (i.e., lacking minute punctures), punctures on metanotum similar to those on mesoscutellum but denser. Mesepisternum with flat-bottomed punctures, punctures coarser to those on pronotum posterodorsally, smooth anteroventrally; border between posterodorsal and anteroventral parts distinct, without epicnemial carina. Dorsal part of metapleuron with several strong striae, ventral part with small, sparse, shallow punctures. Propodeum with very coarse and dense punctures on dorsal part, interspaces between punctures strongly raised to form reticulation, lateral part with transverse striae; posterior surface shiny and largely smooth, with some short oblique striations near median carina apically. Tegula with minute punctures. Metasomal TI with sparse strong punctures, distance between punctures greater than puncture diameter, interspaces with minute punctures; punctures on TII larger and deeper than those on TI, punctures on TIII–V and SII denser than those on TII; TVI and SVI with minute punctures.

**Figure 7.**
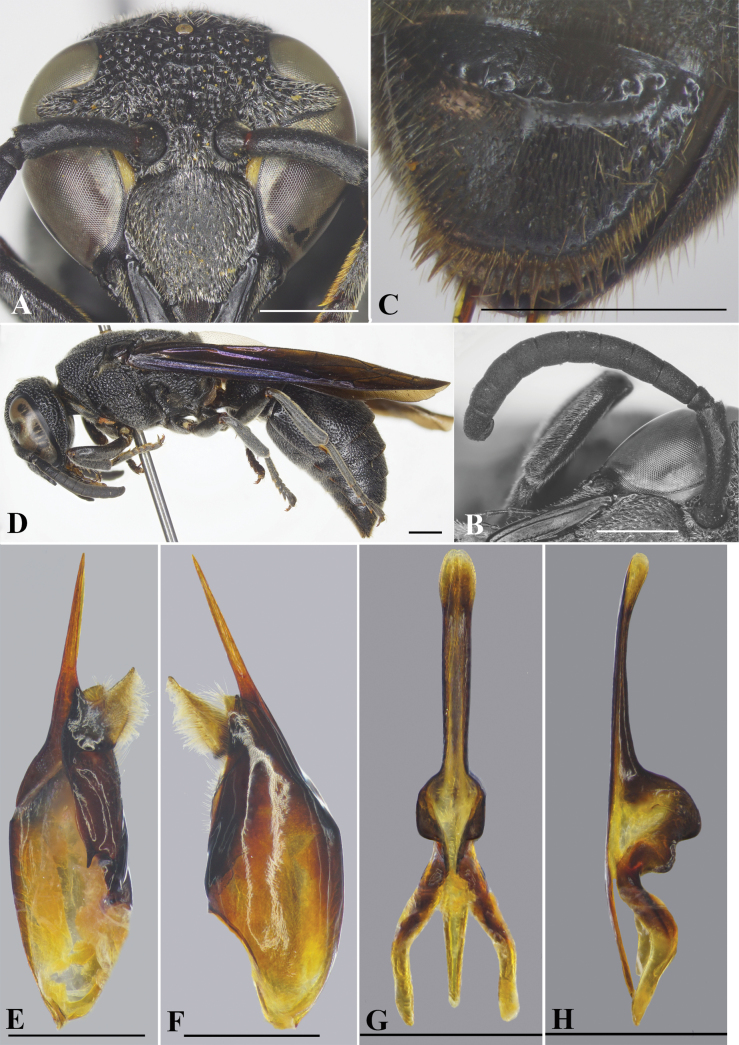
*Allorhynchiummoerum* Nguyen & AD Nguyen, sp. nov., male paratype, female holotype **A** male, facial view **B** male, left antenna **C** male, metasomal sternum VII **D** female, lateral habitus. **E** male genitalia, inner aspect of paramere with volsella and digitus **F** male genitalia, outer aspect of paramere with volsella and digitus. **G** aedeagus, ventral view **H** aedeagus, lateral view. Scale bars: 1 mm.

***Color*.** Body almost black except two yellow bands along compound eyes near clypeus and brown valvulae. Wing strongly infuscate, with purple highlights, veins dark brown (Fig. 7D).

***Pubescence*.** Body with medium-length silver setae.

**Male** (Fig. 7). Body length 11.0–11.7 mm; fore wing length 9.8–11.5 mm.

***Structure*.** As in female but differing as follows: head slightly wider than high, 1.0× as wide as high in anterior view (Fig. 7A); vertex without cephalic foveae; distance from posterior ocelli to apical margin of vertex ~ 2.15× distance from posterior ocelli to inner compound eye margin; inner compound eye margins strongly convergent ventrally, in facial view 1.3× further apart from each other at vertex than at clypeus; clypeus in frontal view almost as wide as long, apical margin deeply emarginate medially, upside down V-shaped, forming sharp pointed tooth on each side (Fig. 7A), width of emargination ~ 1/3 of clypeal width between inner compound eye margins; mandible quadridentate (with four teeth). Antennal scape ~ 3.5× as long as wide, FI ~ 1.6× as long as wide, FII–III longer than wide, FIV–V as wide as long, FVI–VIII wider than long, FX much smaller than FIX, terminal flagellomere elongate, slightly curved, ~ 2× as long as its basal width (Fig. 7A). SVII with a raised flat area basally (Fig. 7B).

***Sculpturing*.** Body surface sculptured as in female; clypeus with coarse but without flat-bottom punctures, distance between punctures greater than in female (Fig. 7A).

***Pubescence*.** As in female except clypeus with dense, long, silver setae.

***Color*.** Similar to female; clypeus usually black but in some specimens, clypeus with two yellow spots subbasally or with one yellow spot medio-apically and a transverse strip basally.

***Genitalia*.** As in Fig. 7 (E–H). Parameral spine lacking hairs. Volsella flattened, spatulate, wide on inner aspect, and with hairs at top (Fig. 7E, F). Digitus gradually widened from base to two-thirds length, then extended apically to form two lobes, a black short one and a transparent lance lobe with dense long hairs (Fig. 7E, F). Penis valves long, much longer than basal apodeme (~ 2.1× as long as basal apodeme); in ventral view proximal part strongly produced laterally into oval shape (Fig. 7G); in profile apical part strongly produced into two large lobes (Fig. 7H), upper lobe larger than lower lobe; proximal margin smooth (Fig. 7H).

#### Distribution.

North and central Vietnam.

#### Etymology.

The specific epithet is from Old Latin *moerus* (= Latin *mūrus*; meaning a wall, a rim, or a dam), and refers to the rim separating the dorsal and posterior surfaces of the propodeum.

#### Remarks.

This new species is similar to *A.chinense* in having the occipital carina being widened laterally; the posterior surface of the propodeum shiny and largely smooth; the propodeal dorsum raised shelf-like to the same level of the metanotum, a deep and wide concavity margined by a crest, and the posterior surface clearly separated from the dorsal and lateral surfaces by a curved rim, the rim produced behind the metanotum to form serrate teeth; SVII of the male with a raised flat area basally; and the forewing strongly infuscate, with purple highlights. However, it differs from the latter by the following characters: body with less coarse punctures (body with coarse punctures in *A.chinense*); pronotum in dorsal view slightly swollen laterally (pronotum in dorsal view strongly swollen laterally); in male, volsella with long setae at the top (volsella without setae at the top in *A.chinense*).

### ﻿ Allorhynchium setosum

Taxon classificationAnimaliaHymenopteraEumenidae

Nguyen & Engel sp. nov.

81074587-0D90-54C9-A34D-8C2E201F1C3E

https://zoobank.org/E8C5A491-FEEC-4FCB-8114-78F642C3A60E

Figs 8 , 9

#### Material examined.

***Holotype*.** Vietnam: ♀, Kon Tum, Sa Thay, Chu Mom Ray NP, 19°47'24.5"N, 104°59'46.5"E, alt. 729 m, 25.iv.2016 [25 April 2016], Lien Thi Phuong Nguyen, Dai Dac Nguyen, Ngat Thi Tran leg. [IEBR].

***Paratypes*.** Vietnam: 1♀, same data as holotype; 1♀, Son La, Phu Yen, Quang Huy, 21°15'50"N, 104°38'56"E, 18.vi.2015 [18 June 2005], Dai Dac Nguyen leg.; 1♀, Son La, Thuan Chau, Long He, Tra May, alt. 1500 m, 3.vi.2009 [3 June 2009], Phong Huy Pham leg.; 1♂, Kon Tum, Dak Ha, Dak Mar, Dak Uy, 14°33'04.6"N, 107°55'08.0"E, alt. 630 m, 19.vii.2021 [19 July 2021], Lien Thi Phuong Nguyen leg.; 1♀, Kon Tum, Sa Thay, Chu Mom Ray NP, 19°47'24.5"N, 104°59'46.5"E, alt. 729 m, 25.iv.2016 [25 April 2016], Lien Thi Phuong Nguyen, Dai Dac Nguyen, Ngat Thi Tran leg.; 1♀, 1♂, Gia Lai, Ka Bang, Konpne, Kon Ka Kinh NP, 14°23'22.9"N, 108°20'27.5"E, alt. 647 m, 15.vii.2012 [15 July 2012], Lien Thi Phuong Nguyen leg.; 1♂, Gia Lai, Ka Bang, Konpne, Kon Ka Kinh NP, 14°11'56.7"N, 108°17'19.5"E, alt. 700 m, 13.vii.2012 [13 July 2012], Lien Thi Phuong Nguyen leg.; 1♀, Gia Lai, Chu Se, rubber and pepper garden, 14.iv.2013 [14 April 2013], Lien Thi Phuong Nguyen leg.; 1♀, Dak Lak, Ea Kar, Ea So, Ea So NR, Station 9, 12°59'15"N, 108°40'18"E, alt. 347 m, 15.iv.2015 [15 April 2015], Lien Thi Phuong Nguyen, Dai Dac Nguyen, Minh Phuong Nguyen leg. [IEBR].

#### Diagnosis.

This species can be distinguished from congeners by the following character combination: vertex of female with a small cephalic fovea bearing a tuft of setae medio-dorsally; occipital carina weakly widen laterally; inner compound eye margins strongly convergent ventrally, in anterior view 1.2× as further apart from each other at vertex as at clypeus; clypeus with apical flattened half well defined, in facial view wider than high; mesoscutum almost as long as wide between tegulae; propodeal dorsum raised shelf-like to same level with metanotum, concavity shallow and narrow; SVII of male with a raised flat area basally, apical margin of raised flat area pointed medially. In male genitalia, digitus extend to apex to form two lobes, a short black lobe smooth laterally, and a transparent wide lobe with dense long setae and several short and thicker setae; penis valves with several setae at basal one-third; proximal part of penis valves in profile with an apical part strongly produced into two large lobes.

#### Description.

**Figure 8.**
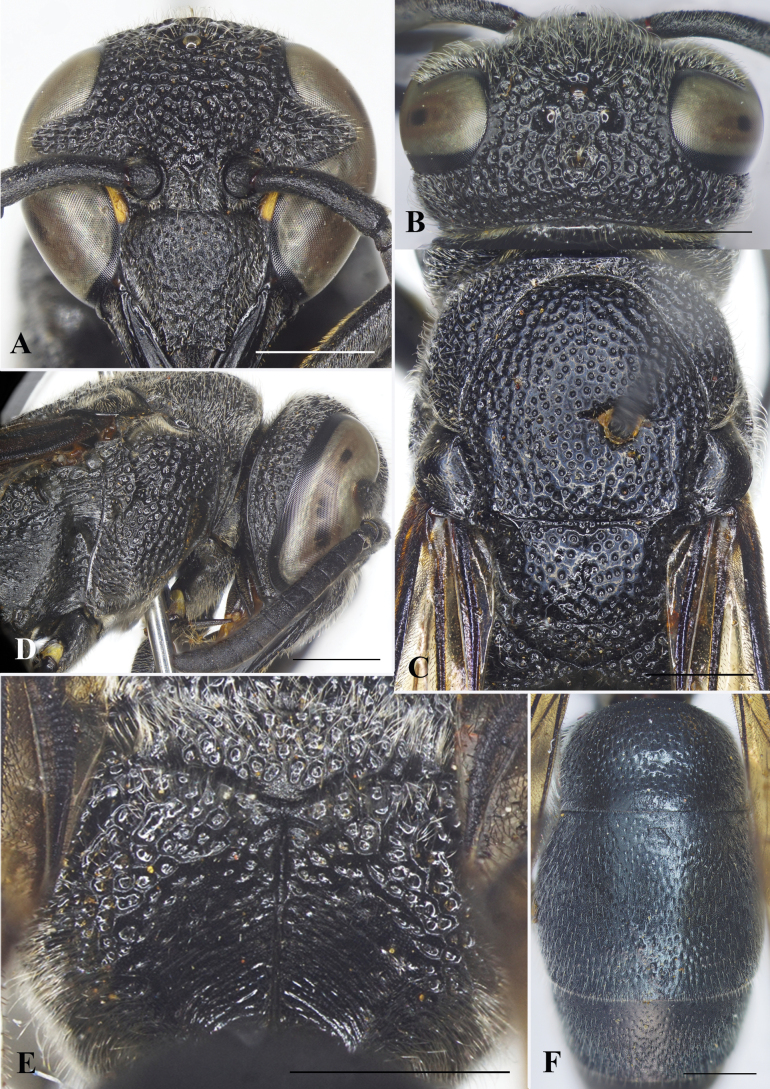
*Allorhynchiumsetosum* Nguyen & Engel, sp. nov., female holotype **A** facial view **B** head, dorsal view **C** mesosoma, dorsal view **D** mesosoma, lateral view **E** propodeum, posterior view **F** metasomal terga I–III, dorsal view. Scale bars: 1 mm.

**Female** (Fig. 8): Body length 10.0–10.7 mm (holotype = 10.5 mm); forewing length 9.5–10.2 mm (holotype = 10.0 mm).

***Structure*.** Head in facial view subcircular, 1.1× as wide as high (Fig. 8A). Vertex with a small cephalic fovea bearing a tuft of setae medio-dorsally (Fig. 8B). Distance from posterior ocelli to apical margin of vertex 1.8× distance from posterior ocellus to inner compound eye margin (Fig. 8B). Gena narrower than compound eye, ~ 0.8× as wide as compound eye; occipital carina complete, present along entire length of gena, weakly widened laterally. Inner compound eye margins strongly convergent ventrally, in anterior view 1.2× further apart from each other at vertex than at clypeus. Clypeus with apical flattened half well defined, in lateral view prominently convex in basal half, then straight to apical margin, in anterior view wider than high, ~ 1.1× as wide as high (Fig. 8A), with basal margin slightly concave medially and distinctly separated from antennal toruli; apical margin shallowly emarginate medially, forming blunt tooth on each lateral side, distance between teeth slightly > 1/3 width of clypeus between inner compound eye margins (~ 0.4× width of clypeus between inner compound eye margins). Mandible quadridentate (with four teeth), teeth prominent. Antennal scape ~ 4.1× as long as its maximum width, slightly curved; FI ~ 1.4× longer than wide, FII slightly longer than wide, FIII–IX wider than long, terminal flagellomere bullet-shaped, ca. as long as its basal width. Mesosoma longer than wide in dorsal view (Fig. 8C). Pronotal carina raised, reaching ventral corner of pronotum. Mesoscutum weakly convex, almost as long as wide between tegulae, without depressed and oblique furrows apically (Fig. 8E). Disc of mesoscutellum slightly convex, in lateral view at same level of mesoscutum, narrowly depressed basally (Fig. 8C). Metanotum weakly convex. Propodeal dorsum raised shelf-like to same level with metanotum; declivity of propodeum with median carina running upward into a narrow and long fovea at short distance from metanotum, concavity shallow and narrow. Forewing with third submarginal cell separated from apex of marginal cell by < ½ its length (Fig. 8D). Metasomal segment I as wide as segment II (Fig. 8F), rounded at base; TI in dorsal view ~ 1.6× as wide as long; TII wider than long, ~ 1.2× as wide as long in dorsal view; SII depressed at base, in lateral view almost straight from base to midlength, then straight to apical margin.

***Sculpturing*.** Clypeus rugose, basal and lateral parts with strong punctures, interspaces between punctures with minute punctures, each puncture bearing a medium-long bristle. Frons densely covered with coarse flat-bottom punctures, interspaces between punctures narrow and raised to form reticulation. Vertex with strong and deep punctures, punctures equal in size, interspaces with sparse minute punctures; gena from midlength to vertex with punctures similar to those on vertex, and with smaller and weaker punctures on apical half (Fig. 8D); occipital carina weakly widened laterally (Fig. 8D). Pronotum with punctures similar to those on vertex. Mesoscutum covered with flat-bottomed punctures, punctures equal in size, smaller than those on pronotum, interspaces between punctures with sparse minute punctures, smooth, larger than puncture diameters centrally, punctures at margins stronger and larger than those centrally; mesoscutellum with punctures similar to those on mesoscutum, punctures on metanotum denser than those on mesoscutellum, interspaces between punctures narrow and raised to form reticulation. Mesepisternum with flat-bottomed punctures, punctures coarser than those on pronotum posterodorsally, smooth anteroventrally; border between posterodorsal and anteroventral parts distinct, without epicnemial carina. Dorsal part of metapleuron with several strong striae, ventral portion of metapleuron with dense and shallow punctures. Propodeum with dorsolateral margin of propodeum somewhat rounded, punctures on dorsolateral area coarse and irregularly rugose, interspaces carinate, sometimes with teeth-like structures behind metanotum, posterior surface rugose basally and with some short oblique striations near median carina apically. Tegula covered with minute punctures. Metasomal TI covered with sparse strong punctures dorsally, and fine punctures dorso-anteriorly, distance between punctures greater than puncture diameter, with minute punctures in interspaces; punctures on TII smaller and shallower than those on TI, punctures on TIII–V and SII denser than those on TII; TVI and SVI with minute punctures.

**Figure 9.**
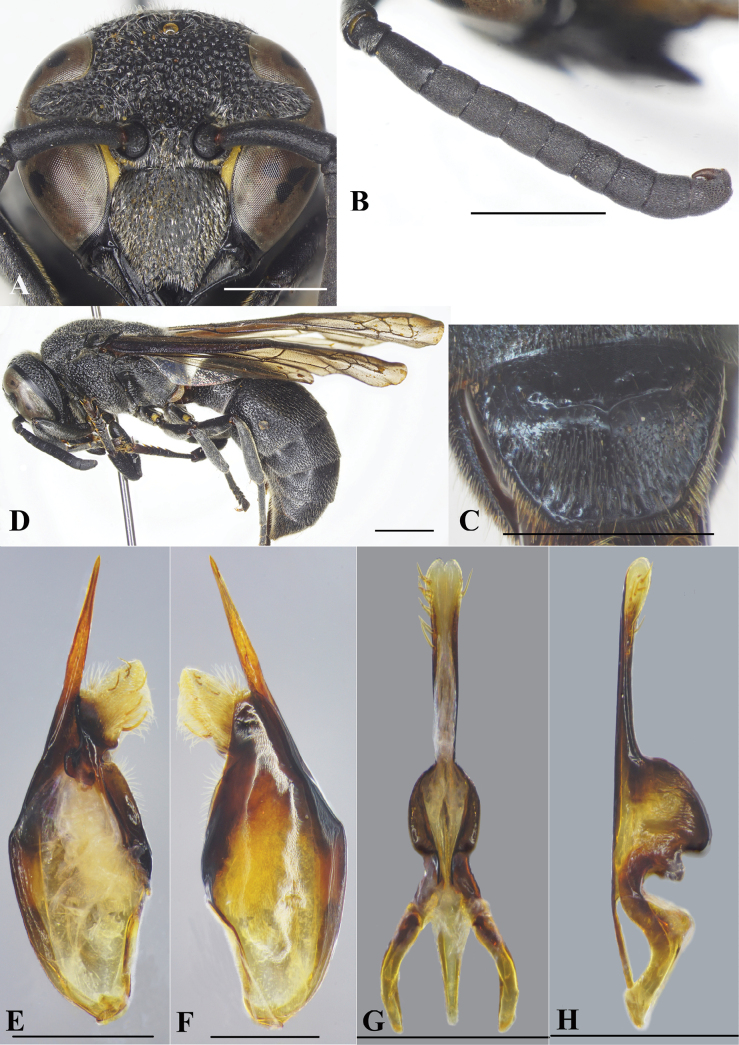
*Allorhynchiumsetosum* Nguyen & Engel, sp. nov., male paratype, female holotype **A** male, facial view **B** male, right antenna **C** male, metasomal sternum VII **D** female, lateral habitus **E** male genitalia, inner aspect of paramere with volsella and digitus **F** male genitalia, outer aspect of paramere with volsella and digitus **G** aedeagus, ventral view **H** aedeagus, lateral view. Scale bars: 1 mm.

***Color*.** Body almost black except two short yellow bands along inner orbits near clypeus and brown valvulae. Wing infuscate, veins dark brown (Fig. 9D).

***Pubescence*.** Body with medium-length silver setae.

**Male** (Fig. 9). Body length 10.4–10.6 mm; fore wing length 9.9–10.1 mm.

***Structure.*** As in female but differing as follows: head wider than high, 1.1× as wide as high in facial view (Fig. 9A); vertex without cephalic foveae; distance from posterior ocelli to apical margin of vertex ~ 2.2× distance from posterior ocelli to inner compound eye margin; inner compound eye margins strongly convergent ventrally, in anterior view 1.3× further apart from each other at vertex than at clypeus; clypeus in anterior view slightly wider than high, apical margin deeply emarginate medially, forming sharply pointed tooth on each side (Fig. 9A), width of emargination greater than one-third of clypeal width between inner compound eye margins (0.4× width of clypeus between inner compound eye margins); mandible with short and wide teeth. Antennal scape ~ 3.6× as long as wide, FI ~ 1.6× as long as wide, FII longer than wide, FIII and FIX as wide as long, FIV–VIII wider than long, FX much smaller than FIX, terminal flagellomere elongate, slightly curved, ~ 3× as long as its basal width (Fig. 9B). Metasomal SVII with a raised flat area basally, apical margin of flat area pointed medially (Fig. 9C).

***Sculpturing*.** Body surface sculptured as in female; clypeus with sparse and small punctures, distance between punctures greater than puncture diameter and with dense minute punctures.

***Pubescence*.** As in female except clypeus covered with dense, medium-long, silver setae.

***Color*.** Similar to female.

***Genitalia*.** As in Fig. 9E–H. Parameral spine lacking setae. Volsella flattened, spatulate, wide on inner aspect, and with long setae at top (Fig. 9E, F). Digitus gradually widened from base to two-thirds length, then extended apically to form two lobes, a dark-brown short one smooth laterally, and a transparent wide lobe with dense long setae and several additional short and thicker setae (Fig. 9E, F). Penis valves long, much longer than basal apodeme (~ 2× as long as basal apodeme), with several setae in basal third; in ventral view proximal part strongly produced laterally into an oval shape (Fig. 9G); in profile apical part strongly produced into two large lobes (Fig. 9H), upper lobe larger than lower lobe; proximal margin smooth (Fig. 9H).

#### Distribution.

North, Central, and Highland of Vietnam.

#### Etymology.

The specific epithet is the Latin adjective *sētōsus* (meaning shaggy), and refers to the setae on the basal third of the penis valves and apex of the digitus (Fig. 9E–H).

#### Remarks.

This new species is closely similar to *A.argentatum* in having the occipital carina slightly widened laterally, the propodeal dorsum raised shelf-like to the same level with the metanotum, the clypeus of the female with a rather deep and wide emargination apically, and the clypeus of the male densely covered with silvery setae. However, it differs from the latter by the following traits: mesoscutum as long as wide between tegulae (mesoscutum wider than long, 1.2× wider than long in *A.argentatum*), mandible of the male with short and wide teeth (mandible of the male with prominent long teeth in *A.argentatum*), SVII of the male with the apical margin of the raised flat area pointed medially (SVII with apical margin of raised flat area wide medially in *A.argentatum*), apex of digitus with two lobes, the short one blunter, the setose transparent lobe much wider and with several short and thick setae (apex of digitus with two lobes, the short one sharper, the setose transparent lance shape and without setae in *A.argentatum*), penis valves with several setae at basal one-thirds (penis valves without setae at base in *A.argentatum*).

This new species is also similar to *A.latum* sp. nov. in having the occipital carina slightly widened laterally, the propodeal dorsum raised shelf-like to the same level with the metanotum, mesoscutum as long as wide between tegulae. But it differs from the latter by the following characters: clypeus wider than high (clypeus as wide as high in *A.latum*), distance between teeth of clypeus < ½ width of clypeus between inner compound eye margins (distance between teeth of clypeus > ½ width of clypeus between inner compound eye margins in *A.latum*), penis valves in profile with lower lobe of apical margin round (penis valves in profile with lower lobe of apical margin produced to a narrow and sharp lobe in *A.latum*).

##### ﻿Key to oriental species of *Allorhynchium*

A key is provided here to the 21 recognized Oriental species in the genus *Allorhynchium*. Some species with the propodeum not level with the metanotum, such as *A.anomalum*, *A.diffinis*, *A.lugubrinum*, *A.quadrimaculatum*, *A.menglianense*, and *A.radiatum*, are tentatively treated under the genus *Allorhynchium*. Further study is needed along with a revision and new key to Oriental eumenine genera so that *Allorhynchium* can be newly redefined and identified properly.

The characters are taken from the descriptions and figures of van der Vecht (1963), Giordani Soika (1986), Yamane (1990), Girish Kumar and Sharma (2015), Girish Kumar et al. (2016), Tan et al. (2018), Li et al. (2019), Luo et al. (2020), except specimens of *A.argentatum, A.latum* sp. nov., *A.lugubrinum*, *A.moerum* sp. nov., *A.quadrimaculatum*, *A.setosum* sp. nov., and *A.snelleni* from Vietnam, *A.argentatum* from Indonesia, *A.chinense* from Taiwan, and *A.metallicum* from India and Malaysia. For the distribution and color of the species refer to van der Vecht (1963), Tan et al. (2018), and Luo et al. (2020).

Because there are some species described based only on the male (such as *A.tuberculatum*, *A.quadrituberculatum*), separate keys are provided for females and males.

### ﻿Key to females

**Table:** 

1	Propodeal dorsum below level of metanotum; metasomal punctures strong	**2**
–	Propodeal dorsum raised shelf-like to same level with metanotum; metasomal punctures weaker	**7**
2	Metasomal segment I with anterior vertical surface clearly separable from posterior horizontal part by a distinct transverse rim; apical margin of clypeus deeply emarginate, emargination almost half-oval shaped	**3**
–	Metasomal segment I with anterior vertical surface smoothly arching to posterior horizontal part, without a transverse rim (except in *A.quadrimaculatum*); apical margin of clypeus more shallowly emarginate	**4**
3	Puncture of TI basally sparse; clypeus with exceptionally coarse punctures, interspaces between punctures smooth and strongly raised to form reticulation	***A.radiatum* Li, Barthélémy & Carpenter, 2019**
–	Puncture of TI basally denser; clypeus vermiculate-reticulate	***A.diffinis* (Giordani Soika, 1986)**
4	Mesoscutum longer than wide in dorsal view (Luo et al. 2020: fig. 5)	***A.menglianense* (Dong & Wang, 2017)**
–	Mesoscutum wider than long in dorsal view	**5**
5	Puncture of TII fine and moderately dense, with interspaces between punctures equal to puncture diameter; SII strongly convex at basal half	***A.anomalum* Giordani Soika, 1992**
–	Puncture of TII coarser and denser, with interspaces between punctures less than puncture diameter; SII gradually convex at basal half	**6**
6	Metasomal segment I with anterior vertical surface clearly separable from posterior horizontal part by a distinct transverse rim; occipital carina greatly widened laterally	***A.quadrimaculatum* Gusenleitner, 1997**
—	Metasomal segment I with anterior vertical surface arching smoothly to posterior horizontal part, without a transverse rim; occipital carina not widened laterally	***A.lugubrinum* (Cameron, 1900)**
7	Pronotal carina forming at most a bluntly rounded angle at shoulders; TI finely punctate dorso-apically	**8**
—	Pronotal carina forming an orthogonal or even sharp, slightly acute angle at shoulders; TI coarsely punctate dorsally	**15**
8	Occipital carina strongly widened laterally (Fig. 6D); posterior surface of propodeum deeply concave, shiny, clearly separated from dorsal and lateral surfaces by a curved rim, rim produced behind metanotum to form serrate teeth	**9**
–	Occipital carina weakly widened or narrow laterally; propodeum without a rim separating dorsal and posterior surfaces	**10**
9	Body with coarse punctures; pronotum in dorsal view strongly swollen laterally	***A.chinense* (de Saussure, 1862)**
–	Body with less coarse punctures; pronotum in dorsal view slightly swollen laterally	***A.moerum* Nguyen & AD Nguyen, sp. nov.**
10	Tomentum and pubescence brownish yellow; scapus and pedicellus, all femora and tibiae, and two large spots on propodeum reddish brown	***A.tigrinum* van der Vecht, 1963**
–	Tomentum and pubescence silver; scapus and pedicellus, all femora and tibiae, and propodeum black	**11**
11	Abdomen with rather fine punctures	***A.metallicum* (de Saussure, 1852)**
–	Abdomen with strong punctures	**12**
12	Wings with diffuse fuscous area apically; apical margins of metasomal segments II–VI covered with fusco-ferruginous pubescence	***A.obscurum* (Smith, 1858)**
–	Wings without diffuse fuscous area apically; apical margins of metasomal segments II–VI covered with silver pubescence	**13**
13	Head transverse, much wider than high; apical margin of clypeus with emargination wide, much > 1/3 width of clypeus between inner compound eye margins	***A.latum* Nguyen, Tran & MT Nguyen, sp. nov.**
–	Head subcircular, slightly wider than high, apical margin of clypeus ~ 1/3 width of clypeus between inner compound eye margins	**14**
14	Punctures on TI and II strong; posterior face of propodeum with shallow concave	***A.argentatum* (Fabricius, 1804)**
–	Punctures on TI and II weaker; posterior face of propodeum with deeper concave	***A.setosum* Nguyen & Engel, sp. nov.**
15	Posterior half of TI and basal half of TII at most sparsely punctulate	***A.violaceipenne* Gusenleitner, 2003**
–	Posterior half of TI and basal half of TII with stronger and denser punctures	**16**
16	Clypeus with narrow V-shaped or circular emargination at apical margin and broad teeth laterally	***A.concolor* van der Vecht, 1963**
–	Clypeus broadly emarginate with narrow teeth laterally	**17**
17	Clypeus truncate apically or nearly so	***A.vollenhoveni* (de Saussure, 1862)**
—	Clypeus with emargination of apical margin trapezoidal, producing two rather sharp teeth laterally (Fig. 3E)	**18**
18	Vertex with two large cephalic foveae, each bearing a tuft of setae; TI ~ 1.5× wider than long; depressed area on SII wide; lateral teeth of propodeum not prominent	***A.cariniventre* Giordani Soika, 1986**
–	Vertex without cephalic fovea; TI shorter, ~ 1.75× wider than long; depressed area on SII narrower; lateral teeth of propodeum prominent	***A.snelleni* (de Saussure, 1852)**

### ﻿Key to males

**Table:** 

1	Propodeal dorsum below level of metanotum; metasomal punctures strong; SVII with two to three tubercules or a pair of flat lobe-shaped protuberances sub-basally	**2**
–	Propodeal dorsum raised shelf-like to same level with metanotum; metasomal punctures weaker; SVII flat or with raised area basally	**7**
2	Metasomal segment I with anterior vertical surface clearly separable from posterior horizontal part by a distinct transverse rim; apical margin of clypeus deeply emarginate, emargination almost half-oval shaped; SVII with three tubercules basally	**3**
–	Metasomal segment I with anterior vertical surface smoothly arching to posterior horizontal part, without a transverse rim (except in *A.quadrimaculatum*); apical margin of clypeus more shallowly emarginate; SVII flat, with two tubercules basally or raised basally	**4**
3	Recurved FXI almost reaching back to basal margin of FIX; apex of penis valve rounded in frontal view (Luo et al. 2020: figs 31, 39); body largely black	***A.radiatum* Li, Barthélémy & Carpenter, 2019**
–	Recurved FXI shorter, with apex not reaching back to basal margin of FIX; apex of penis valve not rounded in frontal view (Luo et al. 2020: fig. 22); body largely yellow	***A.diffinis* (Giordani Soika, 1986)**
4	Mesoscutum longer than wide in dorsal view; recurved FXI short, with apex reaching midlength of FIX (Luo et al. 2020: fig. 3)	***A.menglianense* (Dong & Wang, 2017)**
–	Mesoscutum wider than long in dorsal view; recurved FXI of male longer, with apex reaching back to basal margin of FIX	**5**
5	SVII with apical margin of raised area mediobasally emarginate (Girish Kumar et al. 2016: fig. 12)	***A.anomalum* Giordani Soika, 1992**
–	SVII with two tubercules or flat lobe-shaped protuberances subbasally	**6**
6	Metasomal segment I with anterior vertical surface clearly separable from posterior horizontal part by a distinct transverse rim; occipital carina greatly widened laterally; SVII with two triangular tubercules sub-basally	***A.quadrimaculatum* Gusenleitner, 1997**
–	Metasomal segment I with anterior vertical surface arching smoothly to posterior horizontal part, without a transverse rim; occipital carina not widened laterally; SVII with a pair of flat lobe-shaped protuberances subbasally	***A.lugubrinum* (Cameron, 1900)**
7	Clypeus with a tuberculate projection at subapical margin (Girish Kumar et al. 2016: fig. 3)	***A.tuberculatum* Girish Kumar & Carpenter, 2016**
–	Clypeus without tuberculate projection	**8**
8	Pronotal carina forming at most a bluntly rounded angle at shoulders; TI finely punctate dorso-apically	**9**
–	Pronotal carina forming an orthogonal or even sharp, slightly acute angle at shoulders; TI coarsely punctate dorsally	**16**
9	Occipital carina strongly widened laterally; posterior surface of propodeum deeply concave, shiny, clearly separated from dorsal and lateral surfaces by a curved rim, rim produced behind metanotum to form serrate teeth	**10**
–	Occipital carina weakly widened or narrow laterally; propodeum without a rim separating dorsal and posterior surfaces	**11**
10	Body with coarse punctures; pronotum in dorsal view strongly swollen laterally; volsella without long setae at the top	***A.chinense* (de Saussure, 1862)**
–	Body with less coarse punctures; pronotum in dorsal view slightly swollen laterally; volsella with long setae at the top (Fig. 7E, F)	***A.moerum* Nguyen & AD Nguyen, sp. nov.**
11	Tomentum and pubescence brownish yellow; SVII with subbasal area flat	***A.tigrinum* van der Vecht, 1963**
–	Tomentum and pubescence silver; SVII with subbasal area partly raised	**12**
12	Abdomen with rather fine punctures	***A.metallicum* (de Saussure, 1852)**
–	Abdomen with strong punctures	**13**
13	Wings with diffuse fuscous area apically; apical margins of metasomal segments II–VII covered with fusco-ferruginous pubescence	***A.obscurum* (Smith, 1858)**
–	Wings without diffuse fuscous area apically; apical margins of metasomal segments II–VII covered with silver pubescence	**14**
14	Head transverse, much wider than high; apical margin of clypeus with emargination wide, much > 1/3 width of clypeus between inner compound eye margins; SVII with apical margin of raised flat area V-shaped (Fig. 5C); below lobe of proximal part of aedeagus small and sharp (Fig. 5H)	***A.latum* Nguyen, Tran & MT Nguyen, sp. nov.**
–	Head subcircular, slightly wider than high; apical margin of clypeus ~ 1/3 width of clypeus between inner compound eye margins; SVII with apical margin of raised flat area pointed medially (Fig. 9C) or wide; below lobe of proximal part of aedeagus larger and round (Figs 2I, 9H)	**15**
15	SVII with apical margin of raised flat area wide medially; digitus and penis valves without short and thick setae (Fig. 2 F–I)	***A.argentatum* (Fabricius, 1804)**
–	SVII with apical margin of raised flat area pointed medially (Fig. 9C); digitus apically and penis valves basal one-thirds with several short and thick setae (Fig. 9E–H)	***A.setosum* Nguyen & Engel, sp. nov.**
16	Posterior half of TI and basal half of TII at most sparsely punctulate	***A.violaceipenne* Gusenleitner, 2003**
–	Posterior half of TI and basal half of TII with stronger and denser punctures	**17**
17	Clypeus shallowly emarginate medio-apically; vertex with narrow, transverse, slightly raised impunctate area posterior to ocelli	***A.quadrituberculatum* (von Schulthess, 1913)**
–	Clypeus different shape; vertex without raised impunctate area posterior to ocelli	**18**
18	Clypeus with narrow V-shaped or circular emargination at apical margin and broad teeth laterally	***A.concolor* van der Vecht, 1963**
–	Clypeus broadly emarginate with narrow teeth laterally	**19**
19	Clypeus truncate apically or nearly so	***A.vollenhoveni* (de Saussure, 1862)**
–	Clypeus with emargination of apical margin trapezoidal, producing two rather sharp teeth laterally (Fig. 3F)	**20**
20	TI ~ 1.5× wider than long; depressed area on SII wide; lateral teeth of propodeum not prominent	***A.cariniventre* Giordani Soika, 1986**
–	TI shorter, ~ 1.75× wider than long; depressed area on SII narrower; lateral teeth of propodeum prominent	***A.snelleni* (de Saussure, 1852)**

## Supplementary Material

XML Treatment for Allorhynchium

XML Treatment for Allorhynchium argentatum

XML Treatment for Allorhynchium chinense

XML Treatment for Allorhynchium lugubrinum

XML Treatment for Allorhynchium quadrimaculatum

XML Treatment for Allorhynchium snelleni

XML Treatment for Allorhynchium latum

XML Treatment for Allorhynchium moerum

XML Treatment for Allorhynchium setosum

## ﻿Additional information

### Author ORCIDs

Lien Thi Phuong Nguyen https://orcid.org/0000-0003-3527-9577

Anh D. Nguyen https://orcid.org/0000-0001-9273-0040

Ngat Thi Tran https://orcid.org/0000-0002-7057-4638

Manh Thanh Nguyen https://orcid.org/0009-008-7697-7738

Michael S. Engel https://orcid.org/0000-0003-3067-077X

### Data availability

All of the data that support the findings of this study are available in the main text or Supplementary Information.
